# Electrocortical Dynamics of Usual Walking and the Planning to Step over Obstacles in Parkinson’s Disease

**DOI:** 10.3390/s23104866

**Published:** 2023-05-18

**Authors:** Rodrigo Vitório, Ellen Lirani-Silva, Diego Orcioli-Silva, Victor Spiandor Beretta, Anderson Souza Oliveira, Lilian Teresa Bucken Gobbi

**Affiliations:** 1 Institute of Biosciences, Sao Paulo State University (UNESP), Rio Claro 13506-900, Brazil; 2 Graduate Program in Movement Sciences, São Paulo State University (UNESP), Rio Claro 13506-900, Brazil; 3 Department of Sport, Exercise and Rehabilitation, Northumbria University, Newcastle upon Tyne NE1 8ST, UK; 4 Translational and Clinical Research Institute, Faculty of Medical Sciences, Newcastle University, Newcastle upon Tyne NE2 4HH, UK; 5 School of Technology and Sciences, Sao Paulo State University (UNESP), Presidente Prudente 19060-900, Brazil; 6 Department of Materials and Production, Aalborg University, 9220 Aalborg, Denmark

**Keywords:** gait, locomotion, movement disorders, EEG

## Abstract

The neural correlates of locomotion impairments observed in people with Parkinson’s disease (PD) are not fully understood. We investigated whether people with PD present distinct brain electrocortical activity during usual walking and the approach phase of obstacle avoidance when compared to healthy individuals. Fifteen people with PD and fourteen older adults walked overground in two conditions: usual walking and obstacle crossing. Scalp electroencephalography (EEG) was recorded using a mobile 64-channel EEG system. Independent components were clustered using a k-means clustering algorithm. Outcome measures included absolute power in several frequency bands and alpha/beta ratio. During the usual walk, people with PD presented a greater alpha/beta ratio in the left sensorimotor cortex than healthy individuals. While approaching obstacles, both groups reduced alpha and beta power in the premotor and right sensorimotor cortices (balance demand) and increased gamma power in the primary visual cortex (visual demand). Only people with PD reduced alpha power and alpha/beta ratio in the left sensorimotor cortex when approaching obstacles. These findings suggest that PD affects the cortical control of usual walking, leading to a greater proportion of low-frequency (alpha) neuronal firing in the sensorimotor cortex. Moreover, the planning for obstacle avoidance changes the electrocortical dynamics associated with increased balance and visual demands. People with PD rely on increased sensorimotor integration to modulate locomotion.

## 1. Introduction

Parkinson’s disease (PD) is characterized by the degeneration of dopaminergic neurons of the substantia nigra pars compacta. Consequently, the output nuclei of the basal ganglia become hyperactive and send excessive GABAergic (inhibitory) signaling to the thalamus [1]. It has been shown that PD elicits bursts of activity at the beta band (13–30 Hz) at different regions, which may interfere with the somatosensory control of movements [2]. Moreover, there is a reduction in excitatory signaling from the thalamus to many cortical areas, including the primary motor cortex and primary somatosensory cortex [1]. Then, people with PD show broad cortical dysfunction [3], which includes an overall slowing of cortical activity (e.g., a widespread increase in spectral power in the alpha band as well as a decrease in beta and gamma spectral power) [4]. As previous evidence suggests the involvement of multiple cortical regions in the control of human locomotion [5,6,7,8], PD-related cortical dysfunction may play a role in the walking deficits observed in PD [9,10,11]. Therefore, it is relevant to assess cortical activity during walking tasks in people with PD.

It is well documented that PD impairs walking performance on both level ground (i.e., usual walking) and uneven terrains [12]. Several behavioral studies have reported shortened step length, reduced velocity, increased step-to-step variability, and difficulties in adapting the stepping pattern to accommodate an obstacle in the path during both the approach and crossing phases by people with PD [12,13,14,15]. Due to the significant impact of PD on the neural control of locomotion [10,11], gait impairments and tripping over obstacles have been identified as major causes of falls in PD [16,17]. Therefore, it is necessary to underline the distinct influence of PD on the supraspinal control of locomotion when postural control is challenged to avoid tripping over obstacles.

There are different methods to access brain activity during walking, such as functional near-infrared spectroscopy (fNIRS) and scalp electroencephalography (EEG). Studies investigating fNIRS have reported greater prefrontal cortical activation during usual walking in people with PD compared to healthy older adults [18], or during obstacle avoidance compared to unobstructed walking [18,19,20]. However, fNIRS is not suitable to investigate the rapid changes in brain dynamics required to achieve successful obstacle negotiation during walking. High temporal resolution is possible using scalp EEG. Studies applying mobile EEG have shown that greater balance demands during walking induce reductions in alpha (9–13 Hz) and beta (13–30 Hz) EEG power in sensorimotor cortical areas [7,21,22] in healthy individuals. Further, a recent mobile EEG study demonstrated that healthy young adults present changes in the theta power at the frontal brain region that suggested proactive control when negotiating obstacles [23]. However, only a few studies have applied mobile EEG during locomotor tasks in PD.

PD modifies the electrocortical correlates of control during usual walking and while approaching obstacles. Our recent mobile EEG studies applying single-channel analysis showed an overall slowing of EEG recordings during walking in people with PD [19,20], and condition- (from usual walking to approaching obstacles) and medication-specific modulations. People with PD off medication presented lower gamma power than healthy individuals in the posterior parietal cortex (CPz) while walking and approaching obstacles [20]. This slowing of EEG recordings might represent a physiological marker for the reduction in the excitatory signaling from the thalamus to sensorimotor cortical areas [1], which contributes to gait deficits in PD. Similar findings were obtained by Stuart et al. [24], who observed increased alpha power while walking in people with PD. Levodopa intake increased beta and gamma power (CPz) in both walking conditions [20], suggesting potential effects against the PD-related slowing of EEG recordings. Of particular relevance to the treatment of gait impairments in PD, levodopa-related changes in EEG recordings were associated with levodopa-related changes in gait parameters [20]. In addition, relative to usual walking, people with PD reduced both alpha and beta power in channels corresponding to sensorimotor areas (i.e., FCz, Cz and/or CPz) while approaching obstacles, regardless of their medication state [19,20]. These findings suggest the involvement of alpha and beta reductions to control balance during locomotion. Despite the interesting findings, EEG studies regarding the control of locomotion in PD have been conducted at a single-channel level [9,19,20], limiting the quality of the research outcomes due to difficulties in determining the EEG signal source.

High-density EEG allows the identification of in-brain neural sources of electrocortical dynamics, which present superior quality to describe the supraspinal control of movements [5,25,26]. Extracting power spectral features from neural components related to the electrocortical activity in people with PD during obstacle avoidance is a step forward to better understanding the underlying neural mechanisms of PD-related walking impairments. The power spectral parameters can also be further evaluated by generating specific power ratios, such as the alpha/beta ratio, which has been suggested to indicate aperiodic neural activity, as well as indicate specific neural features in neurological patients [27]. Therefore, the use of power spectrum from in-brain components describing neural sources of electrocortical activity can be a relevant tool for clinical evaluation and may inform the development of enhanced treatment for walking impairments in PD.

The use of high-density EEG can help in unravelling the electrocortical signatures of usual walking and in the planning to avoid an obstacle in PD. Therefore, the aim of this study was to investigate whether people with PD present distinct electrocortical activity in specific brain regions during usual walking and the approach phase of obstacle avoidance when compared to healthy older adults. We first hypothesized that people with PD would present in-brain EEG sources containing greater lower-frequency signals when compared to healthy individuals, corroborating our previous EEG studies applying single-channel-level analysis [19,20]. Second, we hypothesized that adapting the walking pattern to approach an obstacle would reduce alpha and beta EEG power in sensorimotor cortical areas, due to greater demands to modulate balance control [7,21,22]. Moreover, we hypothesized that the obstacle condition would increase beta and/or gamma EEG power in the visual and prefrontal cortices, due to increased visual [28] and cognitive demands [18] of obstacle avoidance relative to usual walking.

## 2. Materials and Methods

### 2.1. Participants and Clinical Assessments

Fourteen healthy older adults and fifteen patients with PD participated in this study (see Table 1 for group details). All individuals gave their informed consent for inclusion before they participated in the study. The study procedures were conducted in accordance with the Declaration of Helsinki, and the protocol was approved by the local Ethics Committee (# 39844814.5.0000.5465). Participants were recruited from our lab database. Patients were selected on the criteria of having a confirmed PD diagnosis from at least one neurologist. The participants of both groups were included if they were able to walk unaided and were community-dwelling. Exclusion criteria included the following: diagnosed major depressive disorder; clinical diagnosis of dementia or other severe cognitive impairment (according to recommendations for utilization of the Mini-Mental State Examination—MMSE—in Brazil; cut-off = 20/24 points for illiterates and those who attended formal education, respectively [29]); chronic musculoskeletal or neurological disease (other than PD). An anamnesis was carried out to rule out conditions and impairments that could interfere with the present experimental procedures and to obtain demographic information (e.g., age, height, body mass, etc.). An experienced movement disorder specialist performed a clinical assessment in order to test people with PD on the Unified Parkinson’s Disease Rating Scale (UPDRS) and the Hoehn and Yahr Rating Scale (H&Y); they were tested in the ON state of medication (approximately one hour after having taken a dose).

### 2.2. Experimental Design and Gait Assessment

Participants walked, at their preferred pace, for 60 s around a 25.8 m oval circuit (with two 6.5 m parallel straights). Two walking conditions were tested: usual walking and obstacle crossing. For the obstacle condition, participants were instructed to step over four foam obstacles (length × width × height: 3 × 60 × 10 cm), evenly spaced along the walking path; this aspect of the protocol was meant to avoid the influence of different inter-obstacle distance on the data as this aspect could add more variability to the study. Four trials for each condition were performed in a random order.

Spatiotemporal gait parameters were recorded by a 5.74 m electronic walkway (200 Hz; GAITRite^®^, CIR Systems, Inc., Franklin, NJ, USA) placed over one straight segment of the circuit. A customized MATLAB algorithm (Mathworks, Natick, MA, USA) was used to calculate the following gait parameters in both conditions (from the GAITRite output): step length, step duration, step velocity, step width (mean of the recorded steps), and step-to-step variability in the same parameters (standard deviation of the recorded steps). For the obstacle condition, the calculated gait parameters referred to the approach phase (i.e., the last four steps before the obstacle), allowing a fair comparison with the usual walking condition.

### 2.3. EEG Recordings and Processing

All EEG signals were recorded using a mobile 64-channel system (eegoTM sports, ANT Neuro, Enschede, The Netherlands), sampled at 1024 Hz. All processing and analyses were performed in MATLAB, using scripts and functions based on EEGLAB 13.0.1b (http://www.sccn.ucsd.edu/eeglab). Initially, individual EEG datasets of both usual walking and walking with obstacle conditions were merged into a single dataset for each participant. The instants of the last heel contact prior to the obstacles were registered as events into the EEG data streaming. For each participant, a similar number of events was randomly created for the usual walking condition (relative to the obstacle condition). The full single datasets were down-sampled to 512 Hz and band-pass filtered (2–50 Hz).

The filtered datasets were screened for the removal of channels exhibiting substantial artifacts following procedures described elsewhere [26]. In general, 50 ± 3 EEG channels were retained after applying all rejection methods (range: 44–55). We re-referenced the remaining channels to an average reference. Subsequently, EEG data sectors presenting exacerbated artifacts (originated from cable movements and/or abrupt head movements) were removed from the continuous EEG datasets. No participants were excluded from the analysis due to EEG artifact issues.

#### 2.3.1. Independent Component Analysis (ICA)

To the cleaned datasets, an infomax ICA was applied to transform the EEG channel data into temporally independent component signals. Approximately 1450 independent components were extracted across all participants. The EEGLAB function ADJUST was applied to objectively define independent components carrying artifacts, such as eye blinks, muscle activity artifacts, and movement-related artifacts [30].

#### 2.3.2. EEG Clustering

The DIPFIT function in EEGLAB was used to model each independent component as an equivalent current dipole within a boundary element head model based on the Montreal Neurological Institute standard brain (MNI, Montreal, QC, Canada). Independent components were removed from further analysis if (1) they were marked as artifactual components in the pre-processing analysis; (2) their best-fit equivalent current dipole accounted for <85% of the variance seen at the scalp and (3) presented locations outside the brain. Therefore, the population of independent components used for the clustering was reduced from ~1450 to 198 across all 29 participants from both groups. In this study, the clustering was performed including both PD and controls as a single group. The rationale is that both patients and controls could present independent components at similar cortical regions, but the electrocortical properties of these components may be group-dependent. The clustering was performed using a k-means clustering algorithm available in EEGLAB on vectors jointly coding similarities in dipole location and scalp topography. The clustering algorithm recommended the creation of 8 clusters, which were maintained for analysis. Four of the eight clusters included independent components from more than seven participants from each group (half of the original group study sample) and were located in cortical areas (Table 2). Thus, we performed all further analyses only on these four clusters of interest.

#### 2.3.3. EEG Absolute Power from Independent Components

For the obstacle condition, the EEG signals were epoched from −2.0 s to 0.5 s, from the last step prior to overcoming the obstacle. The 2.5 s epochs allowed for the acquisition of electrocortical activity during the preparation/planning period prior to obstacle crossing [31], including two full gait cycles prior to overcoming the obstacle, and the step avoiding the obstacle. The same window (−2.0 s to 0.5 s) was used as a baseline. For each participant, a matching number of epochs from the usual walking condition was created for comparison with the obstacle condition. The baseline was removed from the epoched datasets and calculated as the average log spectrum across all epochs from both conditions. The absolute power of the power spectrum from each independent component was subsequently averaged in the theta (5–8 Hz), alpha (9–13 Hz), beta (14–30 Hz), and low gamma (31–50 Hz) frequency bands across the epoch time-course. In addition, the EEG alpha/beta ratio was computed using the absolute power from the alpha and beta bands for each independent component.

### 2.4. Statistical Analysis

For demographic data, unrelated sample Student’s t-tests, Mann–Whitney, and chi-square tests were employed for between-group comparisons. For gait and EEG-dependent variables, two-way ANOVAs (group × condition) were carried out, with repeated measures in the condition factor. The interactions were further assessed with post hoc tests using Bonferroni correction for multiple comparisons. Specifically for the EEG data, the analyses were carried out separately for each cluster. All statistical analyses were run on SPSS for Windows 18.0 and the *p*-value was set to 0.05.

## 3. Results

The two groups were not significantly different in sex, age, body mass, and height (Table 1). People with PD presented mild to moderate disease severity (Table 1). Moreover, people with PD, despite having preserved global cognitive function, obtained lower scores in MMSE than older adults (Table 1).

### 3.1. Gait Parameters

The gait parameters for each group and condition are presented in Table 3. A significant interaction between group and condition was observed for step width. Post hoc tests revealed that the two groups had similar step widths during usual walking conditions, but only people with PD increased the step width in the obstacle condition (*p* < 0.001). No other interactions between factors were observed for gait parameters.

A main effect of group was observed for step length and step velocity. Regardless of the experimental condition, people with PD walked slower and with shorter step length than older adults. A trend of group main effect was observed for step length variability, which was greater for people with PD.

A main effect of condition was observed for step duration, step velocity, step length variability, step duration variability, step velocity variability, and step width variability. Both people with PD and older adults showed greater step duration and step-to-step variability and slower step velocity in the obstacle condition than in the usual walking condition.

### 3.2. Electroencephalography

**Figure 1:**
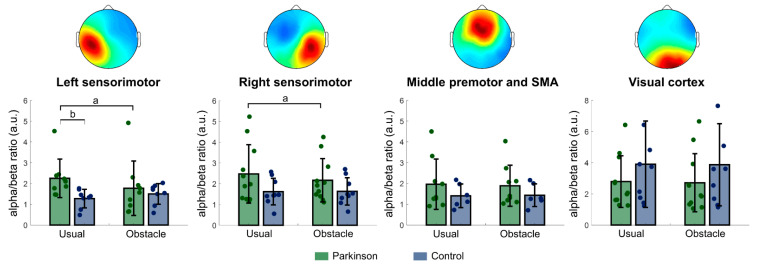
**Top**: Topographic plots for the four clusters identified. **Bottom**: Bar graphs show alpha/beta ratio for people with PD (green) and healthy older adults (blue) in the usual walking and obstacle condition. Circles represent individual values. a indicates significant differences between usual walking and obstacle condition for people with PD; b indicates significant difference between people with PD and healthy older adults.

Four clusters presented independent components with neural characteristics and locations in cortical regions: left and right sensorimotor cortex, supplementary motor area, and primary visual cortex. Table 2 contains details regarding the clusters, and topographic scalp maps of these clusters are shown in Figure 1.

#### 3.2.1. Left Sensorimotor Cortex

**Figure 2:**
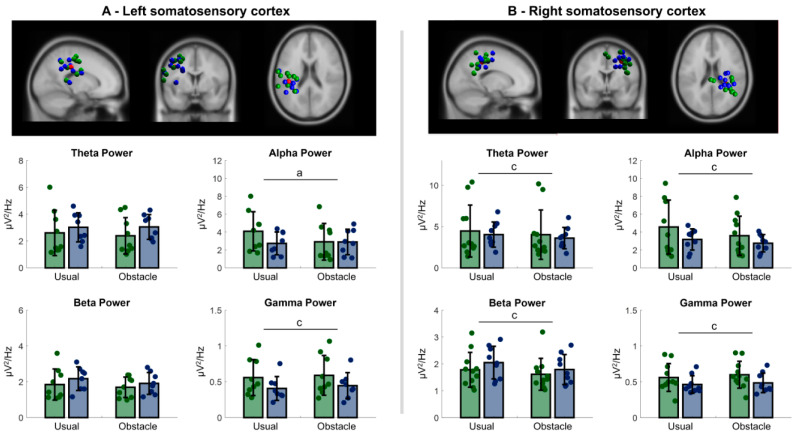
**Top:** Electrocortical clusters of independent components plotted on the MNI brain. Blue spheres represent independent components of healthy older adults and green spheres represent independent components of people with PD. Red spheres represent the centroid locations for the clusters ((**A**)—left sensorimotor cortex; (**B**)—right sensorimotor cortex). **Bottom:** Bar graphs show absolute power for theta, alpha, beta, and gamma bands in the usual walking and obstacle condition. Circles within the graphs represent individual values. a indicates significant difference between usual walking and obstacle condition for people with PD; c indicates main effect of condition.

A significant interaction between group and condition was observed for alpha power [F = 7.974; *p* = 0.013] and alpha/beta ratio [F = 10.501; *p* = 0.005]. Post hoc tests revealed that people with PD showed lower alpha power in the obstacle condition than in the usual walking condition (*p* = 0.002), whereas older adults did not change alpha power across experimental conditions (Figure 2A). Additionally, people with PD showed a greater alpha/beta ratio than older adults in the usual walking condition (*p* = 0.016), and only people with PD decreased alpha/beta ratio in the obstacle condition (*p* = 0.006; Figure 1). A main effect of condition was observed for gamma power [F = 8.509; *p* = 0.011]. Compared to usual walking, both people with PD and older adults increased gamma power in the obstacle condition (Figure 2A).

#### 3.2.2. Right Sensorimotor Cortex

A significant interaction between group and condition was observed for alpha/beta ratio [F = 4.951; *p* = 0.039]; people with PD presented greater alpha/beta ratio in the usual walking condition than in the obstacle condition (*p* = 0.005; Figure 1), whereas older adults did not change alpha/beta ratio across conditions. A main effect of condition was observed for theta [F = 8.179; *p* = 0.01], alpha [F = 14.910; *p* < 0.001], beta [F = 17.910; *p* < 0.001], and gamma power [F = 6.025; *p* = 0.025]. Compared to usual walking, both people with PD and older adults decreased theta, alpha, and beta power and increased gamma power in the obstacle condition (Figure 2B).

#### 3.2.3. Central Premotor and Supplementary Motor Area

**Figure 3:**
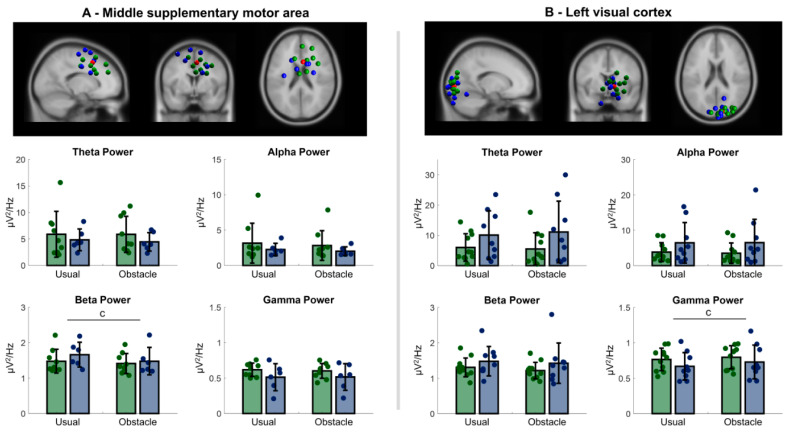
**Top:** Electrocortical clusters of independent components plotted on the MNI brain. Blue spheres represent independent components of healthy older adults and green spheres represent independent components of people with PD. Red spheres represent the centroid locations for the clusters ((**A**)—middle premotor and supplementary motor area; (**B**)—visual cortex). **Bottom:** Bar graphs show absolute power for theta, alpha, beta, and gamma bands in the usual walking and obstacle condition. Circles within the graphs represent individual values. c indicates main effect of condition.

A main effect of condition was observed for beta power [F = 14.623; *p* = 0.002]. Compared to usual walking, both people with PD and older adults decreased beta power in the obstacle condition (Figure 3A).

#### 3.2.4. Primary Visual Cortex

A main effect of condition was observed for gamma power [F = 18.808; *p* < 0.001]. Compared to usual walking, both people with PD and older adults increased gamma power in the obstacle condition (Figure 3B).

## 4. Discussion

The current study investigated whether people with PD present distinct electrocortical activity in specific brain regions during usual walking and the approach phase of obstacle avoidance when compared to healthy older adults. The following findings reveal PD-related changes in electrocortical activity for the control of locomotion: (i) people with PD showed a greater alpha/beta ratio in the left sensorimotor cortex than older adults during usual walking; (ii) people with PD reduced alpha power on the left sensorimotor cortex and alpha/beta ratio on both left and right sensorimotor cortices when approaching obstacles. Overall, these findings support our primary hypothesis and suggest that PD leads to a greater proportion of low-frequency neuronal firing (i.e., relative slowing of scalp EEG) in the sensorimotor cortex that is responsible for motor commands and sensorimotor integration. In addition, our results may suggest that difficulties in integrating sensorimotor inputs to an ongoing ambulatory modulation in the presence of obstacles might help to explain the tripping-related falls experienced by people with PD [32].

### 4.1. PD-Related Changes in the Cortical Control of Locomotion

Activity in the sensorimotor cortex during walking is affected by PD [33,34]. The increased alpha/beta ratio observed in the left sensorimotor cortex in people with PD during usual walking may be related to the excessive GABAergic inhibition of the basal ganglia over the thalamus in PD [1,10]. Among other functions, the thalamus redistributes sensory information and sends excitatory projections to several brain structures, including the primary motor cortex and the somatosensory cortex [1,35,36]. As a consequence of the reduced excitatory activity of the thalamus in PD (due to the excessive inhibitory activity of the basal ganglia over the thalamus), patients’ sensorimotor cortices function with greater proportion of slower waves (e.g., greater alpha/beta ratio) [4]. This may represent the dysfunction of the so called “automatic locomotor network” [37] and/or deficits in sensorimotor integration in PD [30]. Indeed, reduced gait automaticity and an association between sensory deficits and gait impairments have been observed in PD [32].

PD leads to additional electrocortical changes while approaching an obstacle. In the present study, only people with PD reduced alpha power (left) and alpha/beta ratio (left and right) in the sensorimotor cortex in the obstacle condition. Reductions in the alpha power in the somatosensory and primary motor cortex have been associated with movement execution [38,39]. Therefore, we speculate that the relatively greater motor and cognitive demands to avoid obstacles for people with PD induced the desynchronization of additional cortical regions [13,14]. People with PD may rely on increased sensorimotor integration to modulate locomotion and control balance while approaching an obstacle.

### 4.2. Gait and Electrocortical Modulations Required for Obstacle Avoidance

Adjustments in spatiotemporal gait parameters in the approach phase are necessary for successful obstacle avoidance. We observed that both people with PD and older adults decreased their walking speed and increased gait variability prior to overcoming obstacles. Similar findings have been previously reported in the literature and can be interpreted as a strategy for obtaining greater postural stability and having more time to plan the crossing phase [14,19,20]. Moreover, only people with PD increased their step width when approaching the obstacles, corroborating previous studies suggesting that PD imposes additional demands on postural control [40]. This PD-specific step width adjustment also aligns with the observed electrocortical modulations associated with increased balance control.

Reductions in alpha and beta power, previously described in young adults [7,21,22], seem to be involved in balance control while walking in people with PD and healthy older adults. Studies using mobile EEG systems have shown the involvement of both sensorimotor and premotor cortical regions in balance control during different locomotor tasks in young adults. Wagner et al. [21] reported decreased alpha and beta power in the sensorimotor cortex during active walking compared to robot-assisted walking, which reduces balance demand, in young adults. Sipp et al. [7] observed a bilateral reduction in the beta power from the sensorimotor cortex in young adults during walking on a balance beam, which increases balance demand, when compared to treadmill walking. Likewise, Bruijn et al. [22] found decreased beta power in the premotor cortex during the less stable single support phase of gait, and increased beta power in this cortical region during externally stabilized treadmill walking. Our results related to the right sensorimotor and premotor cortical regions are in line with these previous studies. Both healthy older adults and people with PD presented reductions in the alpha and beta power from the premotor and sensorimotor cortices when planning how to overcome obstacles. Such a change in electrocortical activity may be linked to a greater involvement of these brain regions in the monitoring of gait stability prior to engaging in obstacle avoidance.

Processing visual information during the approach phase is particularly relevant for successful obstacle crossing. The activity of the primary visual cortex has been associated with the level of visual attention and perception of visual stimuli. Alpha activity characterizes idle arousal of the system, while beta bursts shift the visual system to an attention state that allows for gamma synchronization and perception [41]. For example, Kaminski and colleagues [42] observed that increased alertness, manifested by faster responses to target visual stimuli, is accompanied by increased beta activity in the visual cortex. This higher beta activity in response to visual stimuli was later associated with preserved cognitive function [43]. In the present study, participants from both groups increased gamma power at the primary visual cortex in the obstacle condition, corroborating a previous study from our research group that evaluated EEG at the channel level [19]. Postural and stepping adjustments to avoid obstacles require greater visual and cognitive engagement [28,44], with it being previously shown that increased attentional demands during gait induced greater changes in gamma activation in several cortical regions [45]. Moreover, obstacle avoidance increases the activation of the prefrontal cortex in both healthy older adults and people with PD [18,20], suggesting increased demands for executive function and attention. Precision stepping tasks have also been shown to induce changes in electrocortical activity in the visual cortex to cope with the postural adjustments to perform non-stereotypical gait [46]. Therefore, the increased gamma power in the primary visual cortex in the obstacle condition suggests a greater need for visual information, and, potentially, the integration of inputs from cognitive, motor, and visual brain regions.

### 4.3. Clinical Implication and Future Directions

Our findings revealed specific PD-related changes in brain dynamics during walking, which may contribute to improving the treatment of walking impairments. Specifically, the greater proportion of low-frequency neuronal firing in the sensorimotor cortex observed in people with PD can be modulated by dopaminergic medication. We have previously shown that levodopa increases beta and gamma power (CPz) during walking [20], suggesting the potential effects of dopaminergic medication against the PD-related slowing of EEG recordings. It is likely that the levodopa-related increases in beta and gamma power (CPz) are due to increased cortical excitability following levodopa intake [47]. Moreover, we have observed that levodopa-related changes in EEG recordings were associated with levodopa-related changes in gait parameters, highlighting specific cortical mechanisms involved in gait improvement [20]. Therefore, high-density EEG outcomes recorded while walking may serve as biomarkers to assess the response to treatment aiming to improve gait in PD [24], but larger studies are needed. Future studies should investigate the effects of other interventions, particularly non-invasive brain stimulation techniques [48,49,50], on sensorimotor EEG outcomes recorded while walking in people with PD. Finally, future studies should consider the effects of disease progression on EEG recordings during walking in PD.

## 5. Conclusions

In summary, our results suggest that PD leads to a greater proportion of low-frequency neuronal firing in brain areas related to motor commands and sensorimotor integration during walking. Moreover, planning to avoid an obstacle changes the electrocortical dynamics associated with increased balance and visual demands in both people with PD and healthy older adults. People with PD rely on increased sensorimotor integration to modulate locomotion and avoid obstacles.

## Figures and Tables

**Table 1:** Demographic characteristics of both groups.

Variables	Parkinson	Control	Statistics
Sex (male/female)	6/9	5/9	X^2^ = 0.056, *p* = 0.812
Age (years)	70.8 (10.5)	70.9 (4.9)	t = −0.02, *p* = 0.984
Body mass (kg)	69.6 (12.4)	69.4 (12.1)	t = 0.046, *p* = 0.964
Height (cm)	162.2 (7.5)	160.8 (8.6)	t = 0.449, *p* = 0.657
MMSE (0–30 score)	27.1 (1.5)	28.7 (1.1)	Z = −2.786, *p* = 0.005 *
UPDRS I (0–16 score)	3.3 (1.9)	-	-
UPDRS II (0–52 score)	8.8 (4.8)	-	-
UPDRS III (0–108 score)	25.8 (9.3)	-	-
Hoehn and Yahr [1/1.5/2/2.5/3]	1/4/5/4/1	-	-

* significant difference between groups; MMSE, Mini-Mental State Examination; UPDRS, Unified Parkinson’s Disease Rating Scale.

**Table 2:** Centroid location for all clusters of electrocortical sources containing independent components from ≥7 participants from the Parkinson’s disease and control groups.

Functional Area	Brodmann	No. of Participants	No. of ICs
(Centroid Location)	Area	(PD/Control)	(PD/Control)
Left sensorimotor cortex	2	9/8	9/8
Right sensorimotor cortex	2	11/9	11/9
Visual cortex	17	11/9	11/9
Central premotor and SMA	6	9/7	9/7

PD, Parkinson’s disease; SMA, supplementary motor area; ICs, independent components.

**Table 3:** Gait parameters of people with Parkinson’s disease (PD) and healthy older adults in both usual walking and obstacle conditions.

Variables	Parkinson		Control		Group		Condition		Group × Condition		
Step	USU	OBT	USU	OBT	F	*p*	F	*p*	F	*p*	Post Hoc
Length (cm)	56.0 (6.6)	55.8 (7.3)	63.3 (6.6)	63.7 (8.2)	8.331	**0.008**	0.058	0.811	0.501	0.485	
Duration (s)	0.52 (0.03)	0.56 (0.03)	0.53 (0.05)	0.56 (0.05)	0.137	0.714	176.968	**0.001**	2.622	0.117	
Velocity (cm/s)	108.4 (14.2)	100.5 (15.5)	120.9 (17.4)	114.9 (17.7)	5.083	**0.032**	57.496	**0.001**	1.035	0.318	
Width (cm)	9.2 (2.5)	10.1 (2.7)	8.6 (1.9)	8.6 (2.1)	1.440	0.241	9.737	**0.004**	11.070	**0.003**	PD: USU < OBT
**Step variability**											
Length (cm)	2.42 (1.01)	6.50 (2.04)	1.93 (0.71)	5.30 (1.49)	4.064	0.054 ^t^	132.582	**0.001**	2.623	0.117	
Duration (s)	0.021 (0.011)	0.080 (0.025)	0.016 (0.004)	0.067 (0.017)	2.972	0.096	267.767	**0.001**	1.353	0.255	
Velocity (cm/s)	6.8 (4.0)	11.2 (2.9)	5.3 (1.5)	11.1 (3.1)	0.682	0.416	71.509	**0.001**	1.601	0.217	
Width (cm)	2.05 (0.62)	2.72 (0.86)	2.04 (0.49)	2.55 (0.60)	0.170	0.684	41.485	**0.001**	0.756	0.392	

USU, usual walking; OBT, obstacle; PD, Parkinson’s disease; *p*-values marked in bold represent statistically significant results.

## Data Availability

All data are available from the corresponding author upon reasonable request.
